# *Pterygodermatites* (*Pterygodermatites*) *mexicana* n. sp. (Nematoda: Rictulariidae), a parasite of *Balantiopteryx plicata* (Chiroptera) in Mexico

**DOI:** 10.1051/parasite/2013047

**Published:** 2013-11-26

**Authors:** Juan Manuel Caspeta-Mandujano, Francisco Agustín Jiménez, Jorge Luis Peralta-Rodríguez, José Antonio Guerrero

**Affiliations:** 1 Laboratorio de Parasitología de Animales Silvestres, Facultad de Ciencias Biológicas. Av. Universidad No. 1001, Col. Chamilpa C.P. 62210 Cuernavaca, Morelos México; 2 Centro de Investigaciones Biológicas, Universidad Autónoma del Estado de Morelos, Av. Universidad No. 1001, Col. Chamilpa C.P. 62210 Cuernavaca, Morelos México; 3 Department of Zoology, Southern Illinois University Carbondale, Illinois 62901-6501 USA; 4 Facultad de Ciencias Agropecuarias, Universidad Autónoma del Estado de Morelos, Av. Universidad No. 1001, Col. Chamilpa C.P. 62210 Cuernavaca, Morelos México; 5 Laboratorio de Sistemática y Morfología, Facultad de Ciencias Biológicas, Universidad Autónoma del Estado de Morelos. Av. Universidad No. 1001, Col. Chamilpa C.P. 62210 Cuernavaca, Morelos México

**Keywords:** Nematode, parasite, *Pterygodermatites*, *Balantiopteryx plicata*, chiroptera, Mexico

## Abstract

A new species of nematode, *Pterygodermatites* (*Pterygodermatites*) *mexicana* n. sp., is described based on specimens recovered from the intestine of the gray sac-winged bat, *Balantiopteryx plicata* (Chiroptera, Emballonuridae), from the Biosphere Reserve “Sierra de Huautla” in the state of Morelos, Mexico. This is the second species in the genus described from bats in the New World, since most of the rictaluriids reported in these hosts belong to the closely related genus *Rictularia* Froelich, 1802. However, members of *Rictularia* possess only a single oesophageal tooth at the base of the buccal capsule, whereas in the current nematodes three conspicuous oesophageal teeth are present. They are therefore included in *Pterygodermatites* Wedl, 1861. The new species is characterized by the presence of 23 small denticles on the periphery of the buccal capsule and by the presence of 40 and 66 pairs of cuticular processes in males and females, respectively. Additionally, males possess 3–4 ventral precloacal fan-like processes, and the cuticular processes of females are divided into 40 pairs of comb-like and 26 pairs of spine-like processes; the vulva opens on the level of approximately pair 40. The dorsally directed stoma and the 40 prevulvar cuticular processes makes it difficult to place the species in any of the subgenera present in the New World, yet characters correspond with the diagnosis of *Pterygodermatites* (*Pterygodermatites*) in the Mediterranean region and North Africa.

## Abstract (fr)

Une nouvelle espèce de nématode, *Pterygodermatites* (*Pterygodermatites*) *mexicana* n. sp., est décrite à partir de spécimens collectés de l'intestin de la chauve-souris *Balantiopteryx plicata* (Chiroptera, Emballonuridae), de la Réserve de la biosphère “Sierra de Huautla” dans l'État de Morelos, Mexique. C'est la deuxième espèce du genre décrite de chauves-souris du Nouveau Monde, puisque la plupart des rictulaires mentionnés dans ces hôtes appartiennent au genre proche *Rictularia* Froelich, 1802. Toutefois, les membres de *Rictularia* ne possèdent qu'une seule dent œsophagienne à la base de la capsule buccale, alors que dans les nématodes décrits ici trois dents œsophagiennes sont présentes et bien visibles. Ils sont donc inclus dans *Pterygodermatites* Wedl, 1861. La nouvelle espèce est caractérisée par la présence de 23 petits denticules sur la périphérie de la capsule buccale et de 40 et 66 paires de processus cuticulaires respectivement chez les mâles et les femelles. En outre, les mâles possèdent 3–4 processus précloacaux ventraux en éventails, et les processus cuticulaires des femelles sont divisés en 40 paires de processus en peigne et 26 paires de processus en épine; la vulve s'ouvre approximativement au niveau de la paire 40. Le stoma dirigé vers le dos et les 40 processus cuticulaires prévulvaires rendent difficile le placement de l’espèce dans l'un des sous-genres présents dans le Nouveau Monde, mais les caractères correspondent au diagnostic de *Pterygodermatites* (*Pterygodermatites*) dans la région méditerranéenne et l’Afrique du Nord.

## Introduction

Most species of rictulariid nematodes (Spirurida) occur in rodents and carnivorous mammals, with relatively few species infecting bats [9]. Characters used in the taxonomy of the group include the number of cuticular subventral processes on the worms, as well as the position of the vulva relative to these processes, the arrangement of caudal papillae and the orientation of the stoma [9]. Species traditionally included in *Pterygodermatites* Wedl, 1861 are characterized by a dorsally displaced stoma, armed with three oesophageal teeth, and by possessing between 29 and 60 pairs of prevulvar spines [5, 8, 9]. To date, only three species of *Pterygodermatites* and seven species of *Rictularia* Froelich, 1802 have been described from bats. Of these, *P. elegans* (Travassos, 1928), *R. lucifugus* Douvres, 1956, *R. macdonaldi* (Dobson, 1880) and *R. nana* Caballero, 1943, have been recorded from bats in the New World [1, 3, 4, 6, 7, 9, 11, 13].

During an investigation of helminth parasites of bats in Mexico, carried out by the research team of the Laboratorio de Parasitología de Animales Silvestres, 26 nematodes of the genus *Pterygodermatites* were recovered from the intestines of the gray sac-winged bat, *Balantiopteryx plicata* Peters, 1867 (Chiroptera: Emballonuridae). A detailed morphological analysis of this material revealed the presence of a new species, which is described below.

## Materials and methods

Between August 2008 and July 2009, 80 specimens of *B. plicata* were collected by placing a mist net across the opening of the abandoned “Mina América”, located in the Biosphere Reserve “Sierra de Huautla”, municipality of Tlaquiltenango, Morelos, México. Bats were killed by cervical dislocation and further subject to a helminthological investigation.

The nematodes recovered from the intestine of the gray sac-winged bats were washed in physiological saline, fixed in hot 4% formaldehyde and cleared in glycerine for microscopic examination. Drawings were made with the aid of a Nikon microscope drawing attachment (Nikon Corporation, Tokyo, Japan). After examination, the specimens were stored in 70% ethanol. Four specimens were dehydrated progressively in a graded ethanol series, dried to a non-liquid state by critical point drying using CO_2_, attached to a SEM stub and sputter coated with gold palladium. Specimens imaged with SEM were exposed to a beam between 3 and 20 kV on a Hitachi S2460 N scanning electron microscope (Hitachi, Ltd., Tokyo, Japan). All measurements are given in micrometres unless otherwise stated. Type specimens and paratypes are deposited in the Colección Nacional de Helmintos (CNHE) at the Instituto de Biología, Universidad Nacional Autónoma de México and in the Colección Parasitológica at the Universidad Autónoma del Estado de Morelos (UAEM), Mexico. Licencia de Colector Científico FAUT – 0211 from Secretaría del Medio Ambiente y Recursos Naturales (SEMARNAT).

## *Pterygodermatites* (*Pterygodermatites*) *mexicana* n. sp.

urn:lsid:zoobank.org:act:C45880BA-2373-4581-B304-201C4C4C6354

Figures 1–26

Type host: *Balantiopteryx plicata* Peters, 1867 (Chiroptera: Emballonuridae)

Type locality: Mexico: Morelos State: municipality of Tlaquiltenango: Mina América (18°27′46.47″N, 99°00′53.45″W).

Site of infection: Intestine.

Prevalence and range of intensity: 20% (16 of 80 bats examined); 1–5 nematodes.

Date of collection: From August 2008 to July 2009. Holotype; allotype and paratypes collected in October 2008.

Etymology: The species is named after its country of origin.

Deposition of type specimens: Holotype, allotype and paratypes, respectively, in the Institute of Biology, UNAM, in Mexico, City (CNHE-8601, 8602, 8603); paratypes in the Faculty of Biology, Parasitological Collection of the Universidad Autónoma del Estado de Morelos, Mexico (COPAUAEM N-450).

### Description

**Figures 1–9:**
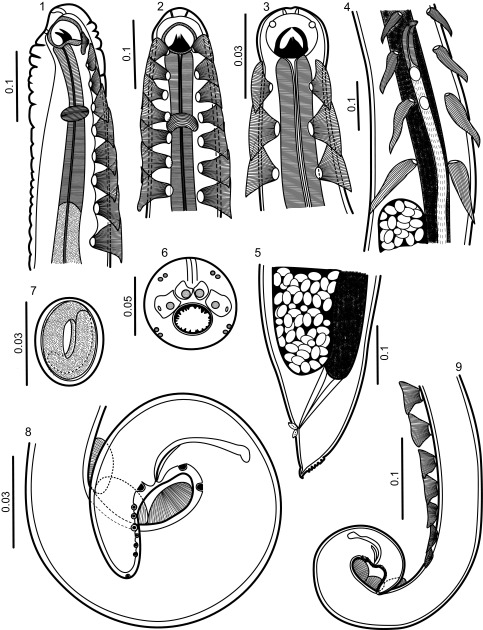
*Pterygodermatites* (*Pterygodermatites*) *mexicana* n. sp. **1.** Anterior end of female, lateral view. **2.** Anterior end of male, ventral view. **3.** Anterior end of female, ventral view. **4.** Region of vulva, ventral view. **5.** Tail of female, lateral view. **6.** Cephalic end of male, apical view. **7.** Embryonated egg. **8, 9.** Tail of male, lateral view.

**General**: Small nematodes with annulated cuticle (Figure 11). Stoma semicircular, subterminal, opens dorsally (Figures 1, 11–14), with small irregularly spaced denticles on margin, 23 in total, 10 dorsal and 13 ventral (Figures 12–14). Well developed sclerotized buccal capsule; three tooth-like projections attached at base (Figures 2–3, 14). Lips not well defined, four lip-like protuberances present on anterior margin of stoma, each bearing one papilla. Four pairs of papillae arranged in an outer circle, two dorsal and two ventral (Figures 6, 11). Amphidial pores present on the lateral lip-like structures (Figures 6, 12–13). Deirids present below the fifth pair of cuticular comb-like processes (hereafter combs) at 275–464 from the anterior end (Figure 16). Tail in both sexes conical (Figures 5, 8, 9, 21–25).

**Figures 10–20:**
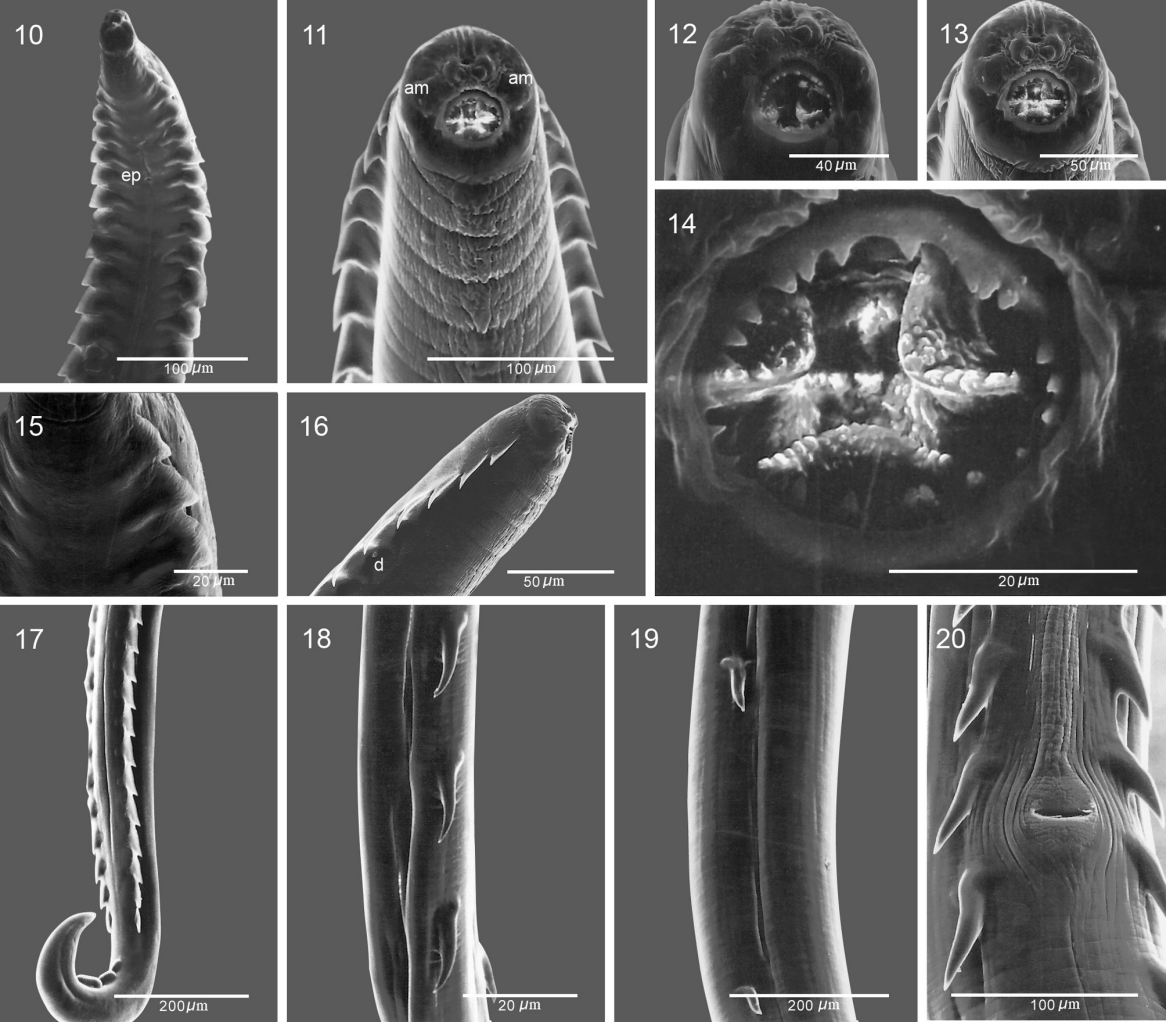
*Pterygodermatites* (*Pterygodermatites*) *mexicana* n. sp. Scanning electron micrographs. **10.** Anterior end of male, ventral view. **11.** Anterior end of female, dorsal view. **12.** Anterior end of male, dorsal view. **13.** Anterior end of female, dorsal view. **14.** Stoma of female, showing denticles. **15.** Ventral view of anterior combs of male. **16.** Anterior end of male in lateral view, showing deirid. **17.** Posterior end of male, showing subventral combs and ventral cuticular processes. **18.** Lateral view of postvulvar subventral spines at midbody of female. **19.** Subventral spines near the posterior end of female. **20.** Spines around the vulva, ventral view. Abbreviations: am-amphid, d-deirid, ep-excretory pore.

**Female** (based on 10 specimens, holotype in parentheses): Length of body, 11.49–20.64 (13.52), maximum width 250–295 (295). Buccal capsule, 40–56 (56) long, measured from ventral rim of capsule to basis, by 43–56 (56) wide. Two rows of subventral cuticular elements extend practically throughout the length of the body; each with 66 elements plus an unpaired comb present on right row. Cuticular elements are more sparse and shorter towards posterior end (Figures 11, 18–20). Cuticular processes show two distinct configurations, combs and spines; pairs anterior to vulva are combs, the rest become spines. Forty paired prevulvar processes plus an unpaired process on right row. Combs 87–131 (95) long by 33–43 (33) wide at basis. Muscular portion of oesophagus 295–350 (295) long, glandular portion 1.81-1.85 (1.85) long. Distance of nerve ring, excretory pore and deirids, 237–250 (246), 311–319 (315) and 300–460 (350) from anterior end, respectively. Vulva preequatorial, 2.41–2.58 (2.58) from anterior end (Figures 4, 20). Eggs oval or almost rounded, 31–42 by 24–31 (31–32 by 25–26), containing first-stage larva (Figures 7, 26). Anal opening with cuticular flanges (Figures 24–25). Tail length 87–116 (112).

**Figures 21–26:**
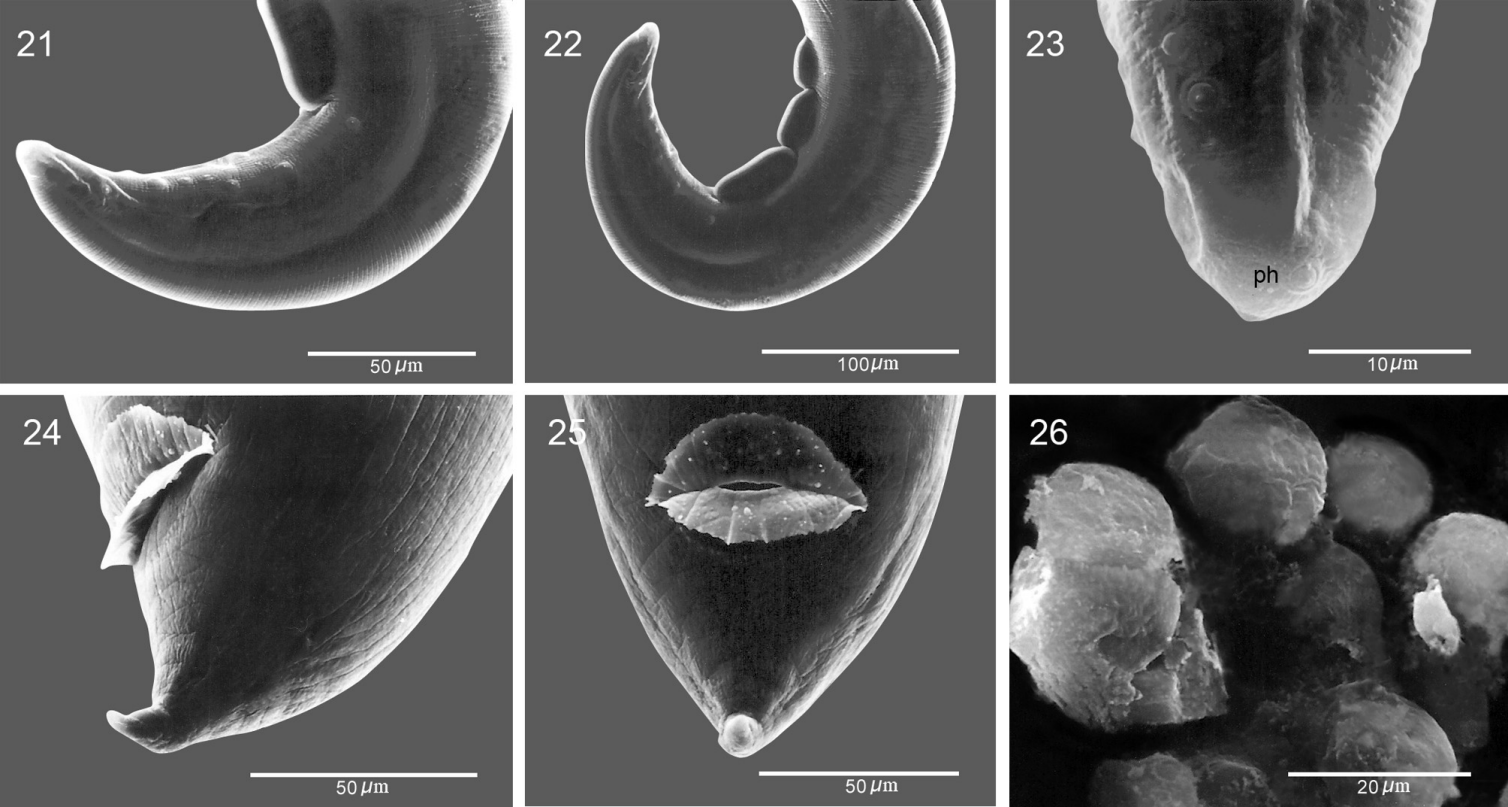
*Pterygodermatites* (*Pterygodermatites*) *mexicana* n. sp. Scanning electron micrographs. **21.** Lateral view of the tail of male showing papillae and posteriormost cuticular ventral process. **22.** Posterior end of male, lateral view. **23.** Lateral view of tail tip of male. **24, 25.** Tail tip of female, lateral and ventral view, respectively. **26.** Egs. Abbreviations: ph-phasmid.

**Male** (based on 10 specimens, allotype in parentheses): Length of body, 1.85–3.83 (2.25), maximum width 75–158 (75). Buccal capsule 13–22 (13) by 18–25 (18) wide. Two subventral rows of combs including 40 pairs, with sharp points projecting posteriad; unpaired process on right row. Length of combs 58–95 (58), width at base 29–39 (30). Muscular portion of oesophagus 150–225 (150) long; glandular portion 462–625 (462) long. Distance of nerve ring, excretory pore and deirids, 156–187 (156), 319–330 (319) and 275–310 (310) from anterior end, respectively. Spicules curved and unequal, left 30–50 (36) long with tapering distal end, right 83–111 (83) long, with tapering distal end. Gubernaculum absent. Three or four fan-like cuticular processes, anterior to cloacal opening (only 1 of the 10 specimens with 4 fan-like cuticular processes), 42–72 (50) long, by 10–29 (20) wide (Figure 22). Nine pairs of caudal papillae plus a terminal pair of phasmids. Two pairs of papillae precloacal, 7 postcloacal; pairs 1, 2, 4, 8 and 9 are sublateral and the remaining subventral (Figures 21–23). Tail conical, 62–116 (62) long (Figure 22).

### Remarks

*Pterygodermatites* (*Pterygodermatites*) *mexicana* can be discriminated from the other species occurring in the New World by the position of the stoma, the number of teeth on the edge of the buccal capsule, the number of cuticular processes in both males and females, and the position of the vulva relative to the cuticular processes. A unique feature of *P.* (*P.*) *mexicana* seems to be the position of the stoma, being almost entirely dorsal (Fig. 16). In addition, the cuticular outgrowth of the anal lips appears to be a unique feature (Figures 24, 25). The inclusion of the species in the subgenus is proposed based on four sets of characters: the dorsally oriented stoma; the presence of irregularly-spaced small denticles in the buccal cavity; the presence of 40 prevulvar cuticular processes, and the sublateral orientation of caudal papillae pairs 1, 4 and 8.

Only three species of *Pterygodermatites* are known to infect bats worldwide, namely *Pterygodermatites* (*Neopaucipectines*) *bovieri* (Blanchard, 1866), *P.* (*Paucipectines*) *elegans* and *P.* (*Pterygodermatites*) *spinosa* (Willemoes-Suhm, 1869). Of these only *P.* (*Paucipectines*) *elegans* occurs in the New World. *Pterygodermatites* (*P.*) *mexicana* can be easily differentiated from *P.* (*Paucipectines*) *elegans* in the position of the stoma, which is apical in the latter [13] and dorsal in the species herein described. In addition, *P.* (*Paucipectines*) *elegans* has been reported exclusively in South America infecting both bats and marsupials [2, 13, 14].

*Pterygodermatites* (*P.*) *mexicana* can be discriminated from *P.* (*P.*) *spinosa* by the number of cuticular elements. The new species features a total of 40 pairs plus an unpaired comb on the right in males; females feature a total of 66 pairs plus an unpaired comb on the right. In *P.* (*P.*) *spinosa* there are 43–44 and 77 in males and females, respectively [10]. *Pterygodermatites* (*P.*) *spinosa* has a Palearctic distribution and has been recorded in the whiskered bat *Myotis mysticinus* (Kuhl, 1817) (Chiroptera: Vespertilionidae) in Germany [9, 10]. The third species, *P.* (*N.*) *bovieri*, infects vespertilionid bats and occurs in localities from Western Europe to Afghanistan [9, 12]. *Pterygodermatites* (*N.*) *bovieri* and *P.* (*P.*) *mexicana* are similar in the number of cuticular processes in both males and females and the arrangement of caudal papillae [12]. However, differences between these two species include the number of teeth surrounding the buccal capsule, and the position of vulva relative to the cuticular processes. A maximum of 14 teeth surround the buccal capsule of *P.* (*N.*) *bovieri* compared to 23 in *P.* (*P.*) *mexicana*; in addition, there are 34 prevulvar processes in *P.* (*N.*) *bovieri* compared to 40 in *P.* (*P.*) *mexicana*.

Finally, irrespective of host and subgenera, *P.* (*P.*) *mexicana* can be discriminated from any other species in the genus occurring in North America by the position of the stoma, which opens dorsally, the number of denticles surrounding the buccal capsule (23), the markedly unequal spicules, the presence of 40 pairs of combs in males and the number of prevulvar processes. Several of the species in the genus showing peribuccal denticles recorded in North America infect rodents of the families Cricetidae, Heteromyidae and Sciuridae [8, 9]. In these species, the stoma opens in an apical position, the spicules are more or less equal and the number of denticles and cuticular processes differs.

In particular, there are seven species that show a markedly different combination of characters and those include: *P.* (*Paucipectines*) *coloradensis* (Hall, 1916), parasite of the Colorado chipmunk *Tamias quadrivittatus* (Say, 1823) (Sciuridae) and the deer mouse *Peromyscus maniculatus* (Wagner, 1845) (Cricetidae), features 14–20 denticles, a total of 42 pairs of cuticular processes in males and less than 31 prevulvar pairs of cuticular processes in females. *Pterygodermatites* (*Paucipectines*) *dipodomis* (Tiner, 1948), parasite of kangaroo rats (Heteromyidae) in the American southwest, featuring 38–40 pairs of cuticular processes in males, and 40 prevulvar pairs of cuticular processes in females. *Pterygodermatites* (*Paucipectines*) *microti* (McPherson and Tiner, 1952), parasite of voles (Cricetidae) in Alaska, featuring no more than 26 peribuccal denticles, 45 pairs of cuticular processes in males and less than 33 pairs of prevulvar cuticular processes. *Pterygodermatites* (*Paucipectines*) *ondatrae* (Chandler, 1941), parasite of muskrats (Cricetidae) in southern United States, featuring no more than 52 pairs of cuticular processes in males, and no more than 32 prevulvar pairs of cuticular processes. *Pterygodermatites* (*Paucipectines*) *onychomis* (Cuckler, 1939), parasites of cricetids and sciurids, featuring no more than 26 denticles and less than 33 prevulvar pairs of cuticular processes. *Pterygodermatites* (*Paucipectines*) *parkeri* Lichtenfels, 1970, parasite of sciurid and cricetid rodents in the east of the United States, featuring a buccal capsule armed with 14–19 denticles, 42 pairs of cuticular processes in males and less than 32 prevulvar pairs of cuticular processes. Finally, *P.* (*Paucipectines*) *peromysci* Lichtenfels, 1970, parasite of sciurid and cricetid rodents in the eastern half of the United States, feature 16–19 denticles in the buccal capsule, a total of 41 pairs of cuticular processes in males and less than 32 prevulvar pairs of cuticular processes; males of this species and *P.* (*Paucipectines*) *dipodomis*, have unequal spicules of similar dimensions as those seen in *P.* (*P.*) *mexicana*, nevertheless, the gubernaculum in the latter has not been seen and the stoma opens dorsally.

## Discussion

The gray sac-winged bat, *Balantiopteryx plicata*, is restricted to areas in the Pacific coast from Costa Rica to Mexico. In Mexico, the Central Neovolcanic Axis roughly acts as a northernmost boundary for this species. The gray sac-winged bat serves as host for both *P.* (*P.*) *mexicana* and *Rictularia nana*, and both species have been described based on material collected in localities in the Central Neovolcanic Axis separated by some 60 km. The two species belong to different genera, however, because there are three tooth-like projections emerging from the base of the buccal capsule of *P.* (*P.*) *mexicana* compared to a single tooth in *R. nana*. In addition, the number and arrangement of caudal papillae in males consists of seven pairs of subventral papillae in *R. nana* and nine pairs of subventral and subventral papillae in *P. mexicana*. The dorsal position of the stoma is a trait considered of valuable taxonomic relevance in the discrimination between both genera, however, Tkach and Swiderski [12], demonstrated that this feature is somewhat variable in bat-dwelling species of *Pterygodermatites.*

Individuals of *P.* (*P.*) *mexicana* and *R. nana* can be easily distinguished by the number of cuticular processes in males, which is 44 pairs in the latter and 40 pairs (plus the single process) in *P.* (*P.*) *mexicana*. In addition, both spicules are shorter in *P.* (*P.*) *mexicana* (30–50 and 83–111) compared to those in *R. nana* (57 and 122). Finally, and perhaps because of the lesser number of cuticular processes, the deirids are located at the level of the fifth pair of combs in *P. mexicana*, whereas these structures are located at the level of the eight pair in *R. nana*.
